# Prediction of Marine Pycnocline Based on Kernel Support Vector Machine and Convex Optimization Technology

**DOI:** 10.3390/s19071562

**Published:** 2019-03-31

**Authors:** Jiachen Yang, Lin Liu, Linfeng Zhang, Gen Li, Zhonghao Sun, Houbing Song

**Affiliations:** 1 School of Electrical and Information Engineering, Tianjin University, 92 Weijin Road, Tianjin 300072, China; yangjiachen@tju.edu.cn (J.Y.); liulinll@tju.edu.cn (L.L.); zhanglinfeng@tju.edu.cn (L.Z.); sunzhonghao@tju.edu.cn (Z.S.); 2 Department of Electrical, Computer, Software and Systems Engineering, Embry-Riddle Aeronautical University, Daytona Beach, FL 32114, USA; h.song@ieee.org

**Keywords:** pycnocline, kernel support vector machine, machine learning, classification, prediction

## Abstract

With the explosive growth of ocean data, it is of great significance to use ocean observation data to analyze ocean pycnocline data in military field. However, due to natural factors, most of the time the ocean hydrological data is not complete. In this case, predicting the ocean hydrological data by partial data has become a hot spot in marine science. In this paper, based on the traditional statistical analysis literature, we propose a machine-learning ocean hydrological data processing process under big data. At the same time, based on the traditional pycnocline gradient determination method, the open Argo data set is analyzed, and the local characteristics of pycnocline are verified from several aspects combined with the current research about pycnocline. Most importantly, in this paper, the combination of kernel function and support vector machine(SVM) is extended to nonlinear learning by using the idea of machine learning and convex optimization technology. Based on this, the known pycnocline training set is trained, and an accurate model is obtained to predict the pycnocline in unknown domains. In the specific steps, this paper combines the classification problem with the regression problem, and determines the proportion of training set and test formula set by polynomial regression. Subsequently, the feature scaling of the input data accelerated the gradient convergence, and a grid search algorithm with variable step size was proposed to determine the super parameter c and gamma of the SVM model. The prediction results not only used the confusion matrix to analyze the accuracy of GridSearch-SVM with variable step size, but also compared the traditional SVM and the similar algorithm. At the end of the experiment, two features which have the greatest influence on the Marine density thermocline are found out by the feature ranking algorithm based on learning.

## 1. Introduction

Pycnocline has always been one of the focuses of physical oceanography. The characteristics of pycnocline relate to water masses, circulation, and internal waves in the ocean [1]. At the same time, the characteristics of pycnocline have an effect on underwater acoustic communication, detection, monitoring, submarine warfare and so on in the military field [2]. Therefore, the study of marine pycnocline is of great value in the field of marine hydrology and military affairs.

The formation of pycnocline is mainly affected by seawater temperature and salinity. Usually, when the wind is calm, the water temperature decreases as the depth increases. When there is a large wind wave, the sea water is mixed up and down, the upper water temperature is gradually uniform, and the temperature of the lower ocean current, which can not be affected by the wind wave, is still decreasing. As a result, the sea water temperature between the upper and lower layers changes dramatically, resulting in the formation of pycnocline. Besides temperature, salinity also plays an important role in the formation of marine pycnocline [3]. For example, where a warm current flows through, the solubility of salt is higher and the salinity is higher, the density of the area will increase [4]. As a general rule, in the vicinity of rivers entering the sea, fresh water desalinating the sea water, which reduces the salinity of the sea water, it also leads to the sudden change of sea water density, thus it is easy to form pycnocline at the boundary between fresh water and sea water [5].

In order to study ocean pycnocline, we need to get a lot of accurate ocean data. However, due to the breadth of the ocean area and the unpredictable nature of the deep sea, it is very difficult for human beings to detect in the sea water [6]. The data obtained by traditional underwater sensors only involve scalar data such as temperature, pressure, depth and so on. For practical applications, the types of observation data fall far short of the requirements [7]. So it’s becoming more and more necessary to analyze existing hydrological data and get new unknown data [8,9]. Through the prediction and analysis of ocean data, we can also improve the existing model and improve the accuracy of data analysis [10,11,12].

On the other hand, with the increasing scale and complexity of ocean hydrological data, the traditional physical statistical model is not robust. Jiang’s article on “A Machine Learning Approach to Argo Data Analysis in a Thermocline” gives a good example of combining the statistical model with the prediction model, which analyses the historical data of the thermocline [13]. The machine learning algorithm is used to find out the most important factors affecting thermocline. This suggests that we can maximize the relationship between ocean hydrological elements (water speed, temperature, salinity, depth, density) under a limited sample training set using the machine learning correlation algorithm [14]. This is of great significance for predicting the distribution characteristics of ocean pycnocline.

**Figure 1:**
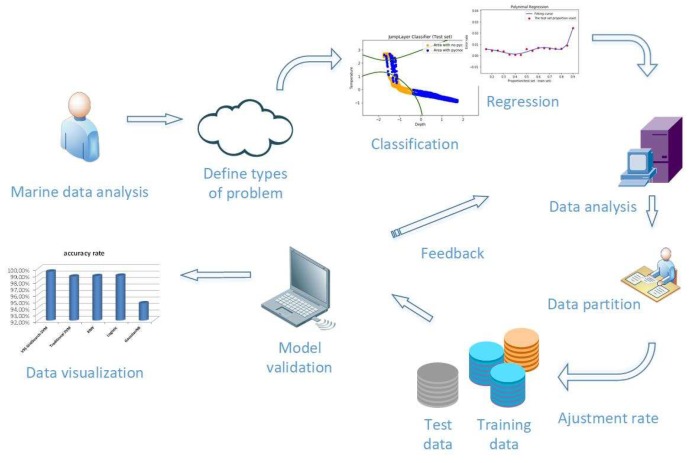
Ocean hydrological data processing model based on machine learning.

The prediction of ocean hydrological data based on machine learning algorithm is different from the general physical statistical model. The following Figure 1 shows the ocean hydrological data process based on machine learning:

The innovations of this article are as follows: 1, We use the ocean density algorithm to calculate the density of the original temperature and salt data, and extract the features of the original open source Argo data structure. Feature extraction is to reduce the dimension, thus reducing the computational complexity. Then we structured the data set after feature extraction and stored it. 2, On the basis of traditional statistical analysis, this paper also analyzes the pycnocline distribution characteristics after calculation, and verifies the characteristics of pycnocline under the influence of time, space, pressure, temperature, salinity and so on. 3, In the experimental part of machine learning, the proportion of the training set is selected by polynomial regression, so as to ensure that the prediction model will not be overfitted to the maximum extent. 4, Subsequently, the feature scaling of the input data accelerated the gradient convergence, and a grid search algorithm with variable step size was proposed to determine the super parameter c and gamma of the SVM model. 5, The prediction results not only used the confusion matrix to analyze the accuracy of GridSearch-SVM with variable step size, but also compared the traditional SVM and the similar algorithm. 6, At the end of the experiment, two features which have the greatest influence on the Marine density thermocline are found out by the feature ranking algorithm based on learning.

The next part is divided into the following parts: in the second part, the related knowledge and characteristics of pycnocline are briefly described. What’s more, the distribution characteristics of pycnocline are analyzed by using the data collected by the open Argo buoy, and the ocean density algorithm is introduced. At the same time, the paper explains why it is necessary to use kernel support vector machine algorithm to predict marine pycnocline. In the third part, the existing ocean data is used to model, and the prediction model of pycnocline is obtained.

## 2. Related Work

In this part, we will enumerate the ocean density algorithm and analyze its calculation process. In addition, we will also expound and explain the causes of formation for the Marine pycnocline and the classification of the pycnocline. At the same time, we will give the ocean data analysis structure based on machine learning kernel-SVM.

### 2.1. A Formula for Calculating Ocean Density

Studies of the density of oceans have yielded preliminary achievement for decades. According to the “Background papers and supporting data on the International Equation of State of Seawater 1980” published by UNESCO in marine science and technology, the functional relationship between density and pressure, temperature and salinity is presented in detail [15].

Density on the spot of the ocean $\left(\rho ,\;\mathrm{kg}/m^{3}\right)$ can be represented as:

(1) $$\begin{array}{c} \rho \left(S,t,P\right)=\frac{\rho \left(S,t,0\right)}{1-P/K\left(S,t,P\right)} \end{array}$$

In the formula, S,t,P respectively represent the salinity,temperature,pressure. $\rho \left(S,t,0\right)$ indicates the density of seawater at a standard atmospheric pressure P = 0. At,Bt are the coefficients of salinity in this equation which are varying with time. *C* is a constant.

(2) $$\begin{array}{c} \rho \left(S,t,0\right)=\rho w+At\times S+Bt\times S^{3/2}+C\times S^{2} \end{array}$$

(3) $$\begin{array}{cc} At=\; & 8.24493\times 10^{-1}-4.0899\times 10^{-3}t+7.6438\times \\ & 10^{-5}t^{2}-8.2467\times 10^{-7}t^{3}+5.3875\times 10^{-9}t^{4} \end{array}$$

(4) $$\begin{array}{cc} Bt= & -5.72466\times 10^{-3}+1.0227\times 10^{-4}t-1.6546\\ & \times 10^{-6}t^{2} \end{array}$$

(5) $$\begin{array}{c} C=4.8314\times 10^{-4} \end{array}$$

The ρw is standard average seawater density, which is given by the following formula:

(6) $$\begin{array}{cc} \rho w=\; & 999.842594+6.793952\times 10^{-2}t-9.095290\\ & \times 10^{-3}t^{2}+1.001685\times 10^{-4}t^{3}-1.120083\\ & \times 10^{-6}t^{4}+6.536332\times 10^{-9}t^{5} \end{array}$$

$K\left(S,t,P\right)$ is called the secant volume modulus, which can be expressed as:

(7) $$\begin{array}{c} K\left(S,t,P\right)=K\left(S,t,0\right)+As+{\left(Bs\right)}^{2} \end{array}$$

Secant volume modulus formula: As, Bs, Aw, Bw is a function of time and salinity. $K\left(S,t,0\right)$ denotes the cut volume modulus at standard atmospheric pressure P = 0.

(8) $$\begin{array}{cc} \mathrm{As}= & \mathrm{Aw}+2.2838\times 10^{-3}-1.0981\times 10^{-5}t\\ & -1.6078\times 10^{-6}t^{2}S+1.91075\\ & \times 10^{-4}S^{3/2} \end{array}$$

(9) $$\begin{array}{cc} \mathrm{Bs}=\; & \mathrm{Bw}+-9.9348\times 10^{-7}+2.0816\times 10^{-8}t\\ & +9.1697\times 10^{-10}t^{2}S \end{array}$$

(10) $$\begin{array}{cc} \mathrm{Aw}=\; & 3.239908+1.43713\times 10^{-3}t+1.16092\\ & \times 10^{-4}t^{2}-5.77905\times 10^{-7}t^{3} \end{array}$$

(11) $$\begin{array}{cc} \mathrm{Bw}=\; & 8.50935\times 10^{-5}-6.12293\times 10^{-6}t\\ & +5.2787\times 10^{-8}t^{2} \end{array}$$

(12) $$\begin{array}{c} K\left(S,t,0\right)=kw+Et\times S+Ft\times S^{3/2} \end{array}$$

In the equation for $K\left(S,t,0\right)$, Et,Ft,kw are just equations that change over time.

(13) $$\begin{array}{cc} Et=\; & 54.6746-0.603459t+1.09987\times 10^{-2}t^{2}\\ & -6.1670\times 10^{-5}t^{3} \end{array}$$

(14) $$\begin{array}{cc} Ft=\; & 7.944\times 10^{-2}+1.6483\times 10^{-2}t-5.3009\\ & \times 10^{-4}t^{2} \end{array}$$

(15) $$\begin{array}{cc} kw=\; & 19652.21+148.4206t-2.327105t^{2}\\ & +1.3360477\times 10^{-2}t^{3}-5.155288\times 10^{-5}t^{4} \end{array}$$

### 2.2. Ocean Data Formatting

Using the Argo data set published by China Argo Real time data Center, we use matlab to study the distribution characteristics of pycnocline. The monthly mean temperature and salt standard layer data from 2004 to 2010 are used. The Argo data set is a three-dimensional grid data set, covering a longitude and latitude range of $120^{\circ }E∼70^{\circ }W$, $60^{\circ }S∼60^{\circ }N$. It has a horizontal resolution of $1^{\circ }\times 1^{\circ }$ and a depth range of 0–2000 m.

We selected the data from 2004 to 2010 at $-5.211^{\circ }$ and $-115.653^{\circ }$ longitude. Temperature and salt data with depth interval of about 1 m were obtained by interpolation method published by Reiniger et al. Finally, the density of seawater between 102 $\mathrm{kg}/m^{3}$ and 105 $\mathrm{kg}/m^{3}$, that is, 1.02 $g/cm^{3}$ to 1.05 $g/cm^{3}$, is obtained by the conversion of density to pressure, temperature and salinity [16,17].

We format the above 10 columns (Table 1) of data. Then, according to the density calculation method mentioned above, the data in 12 columns of data format are stored in combination with time and latitude and longitude. Table 1 below contains the raw Argo data type and the collated data type, respectively:

### 2.3. Determination of Pycnocline

For the study of pycnocline, one of the most important steps is to determine the pycnocline. There are many methods used to determine the cline structure in history, including vertical gradient method, maximum curvature point method and pycnocline determination method (S-T method) proposed by Srintall and Tomczak [18,19].

In this paper, according to the vertical gradient method used in “China Marine Survey Code”, the pycnocline is determined. When the water depth is greater than 200 m, the density gradient is greater than or equal to, and when the water depth is less than 200 m, the density gradient is defined as pycnocline [20]. The depth of the upper and lower end of the cline is the depth of the upper and lower boundary of the cline, and the difference between the upper and lower boundary is the depth of the cline [21,22,23].

According to the traditional definition of pycnocline, a layer of water that suddenly changes in vertical density is called pycnocline. We define the density gradient as G, and by definition we can write the relationship between the density gradient G and the density *D*, depth d (or pressure), and the number of layers *n*:

(16) $$\begin{array}{c} G=\frac{D_{n+1}-D_{n}}{d_{n+1}-d_{n}}\;\;\;\;\;n=2,3,4,5\ldots \ldots \end{array}$$

For the upper formula, G may be positive or negative. For pycnocline, to simplify the formula here:

(17) $$\begin{array}{c} G=\left|G\right|>0 \end{array}$$

For a special case, when the computational depth is the first layer (*n* = 1), we will define the density gradient G = 0.

### 2.4. Kernel-SVM Algorithm

Support vector machine (SVM) is an optimal design criterion for linear classifiers proposed by Vapnik et al on the basis of statistical learning theory. It can effectively solve the problem of linear separability [24]. Support vector machine (SVM) is a supervised learning model, which aims to find a hyperplane in the feature space and separate positive and negative samples with minimum error rate [25]. Support vector machines are usually used in pattern recognition, classification and regression analysis [26,27,28].

For the linear inseparability problem, SVM can also use nonlinear mapping algorithm to map the sample space to a high dimensional or even infinite dimensional feature space (Albert space). So that the problem of nonlinear separability in the original sample space is transformed into a problem of high dimensional linear separability [29]. To put it simply, it is to increase and linearize the original problem. However, the dimension elevation can greatly increase the computational complexity, the display expression of nonlinear mapping can not be determined in some situations. How to find the most suitable dimension is the direction of the optimization of SVM algorithm by relevant researchers in the past decade or so [30].

In order to solve the complexity of ascending dimension, kernel support vector machine came into being. By the expansion theorem of kernel function, we can skillfully avoid the kernel function of high dimensional display expression. Kernel support vector machine which must be obtained in three forms: 1, Poly nomial Kernel; 2, Sigmoid Kernel; 3, Gaussian RBF Kernel.

1. Poly nomial Kernel:

(18) $$\begin{array}{c} K\left(X,Y\right)={\left(\gamma \cdot X^{T}Y+r\right)}^{d},\gamma >0 \end{array}$$

2. Sigmoid Kernel:

(19) $$\begin{array}{c} K\left(X,Y\right)=tanh\left(\gamma \cdot X^{T}Y+r\right) \end{array}$$

3. Gaussian RBF Kernel:

(20) $$\begin{array}{c} K\left(\vec{x},\vec{l^{i}}\right)=e^{-\frac{\vec{x}-{\vec{l^{i}}}^{2}}{2\delta^{2}}} \end{array}$$

The purpose of this paper is to find the best prediction model to predict the location of pycnocline with the least amount of data. So how to find the kernel function suitable for ocean pycnocline prediction is very important.

Polynomial kernel function is a common kernel function, which is very suitable for the prediction of orthogonal normalized data. However, polynomial kernel functions often increase the complexity of functions because of the number of determined parameters.

When Sigmoid function is used as kernel function, support vector machine(SVM) implements a multilayer perceptron neural network. Because it finally obtains the global optimal value rather than the local minimum value, it also ensures its good generalization ability to unknown samples without ever learning phenomenon. However, the use of Sigmoid kernel function will also make the calculation of the activation function large, and the gradient will disappear easily when it is propagated back.

In this paper, Gaussian RBF function is used as kernel function. Not only does it have fewer parameters than polynomial kernel function and faster caculation speed than Sigmoid kernel function, but RBF can achieve similar performance to sigmoid [31,32] by setting some parameters At the same time, it also has good anti-interference ability to the noise in the data.

## 3. Experimental Evaluation

**Figure 2:**
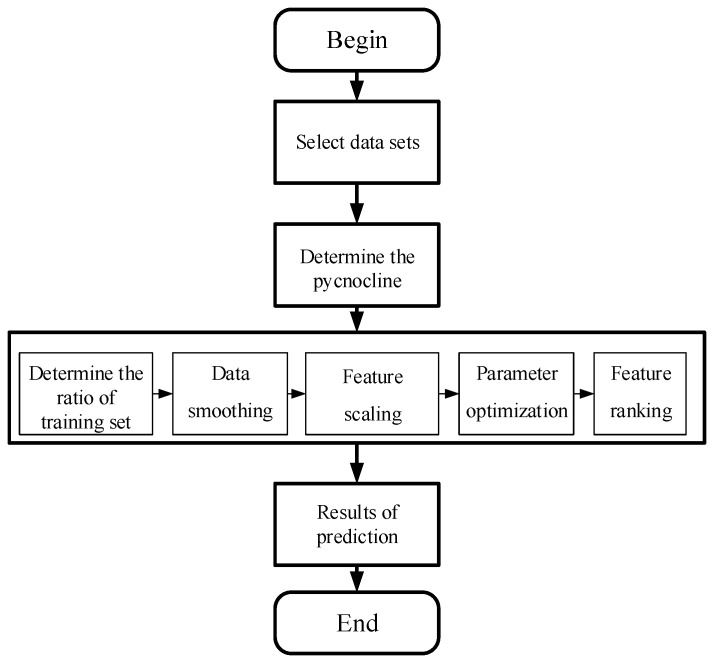
The flow chart of the experiment.

According to the science and technology in recent years, the oceanographic data obtained by the survey ship cannot contain the density in an unknown area of the ocean. Therefore using the known data set to establish a reliable model of pycnocline prediction is the main problem to be solved in this chapter. Since this experiment’s type is supervising learning, the establishment of the prediction model will not include the density data. Finally, the reliability of the prediction model is evaluated by the comparing of the predicted value with the real density data, and the feasibility of the classification algorithm in the prediction of marine data is analyzed. Figure 2 shows the whole process of the experiment.

### 3.1. Selection of Data Sets

**Figure 3:**
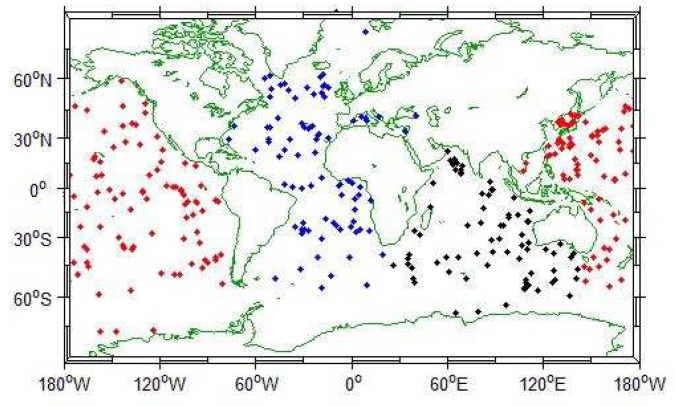
Global Argo collection point distribution.

**Figure 4:**
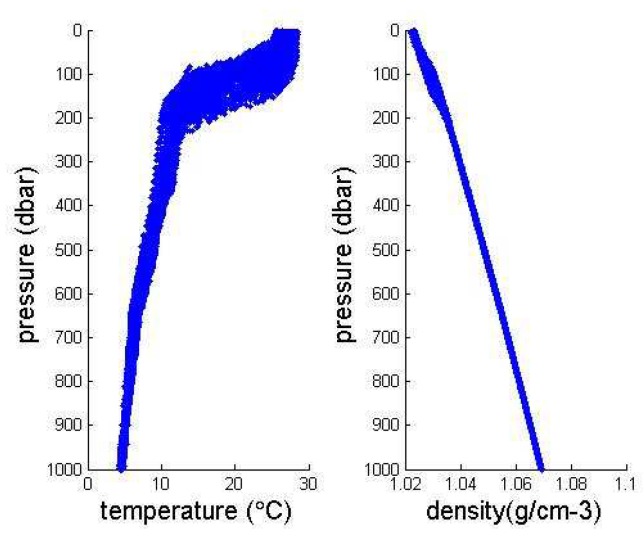
Temperature and density variations with depth over the Pacific.

**Figure 5:**
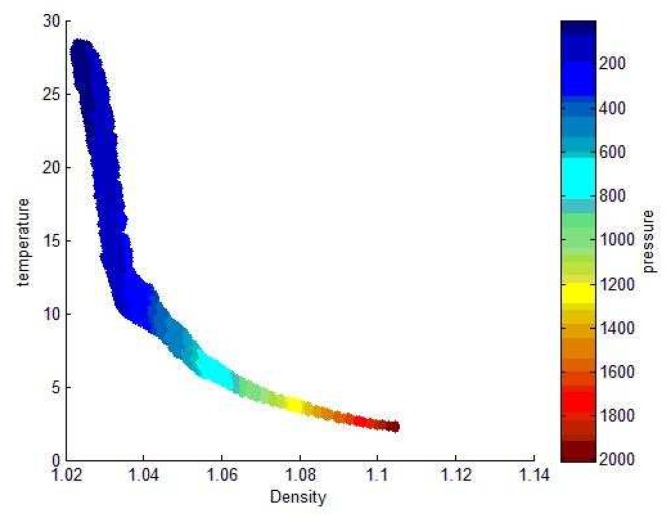
Relationship between density, temperature and depth within the ocean depth range 0–2000 m.

In this section, we use data from the global Argo profile data set, whose Argo distribution is shown in the following figure (Figure 3):

Because of the large data set, we intercept the Argo 3D grid data set published by China Argo Real time data Center from 2004 to 2010, which can be used to predict machine learning. In order to further narrow the range of feature extraction, we need to study the depth range (pressure range) that has the greatest influence on ocean pycnocline. Then we plotted the relationship between density and temperature with depth in the range of 0–1000 m and 0–2000 m, as shown in the following Figure 4 and Figure 5:

It can be seen from the image that the density of the data with a depth of less than 400 m and a temperature above $7^{\circ }$ is usually below 1.04. The depth of 1200–2000 m and the temperature below $3^{\circ }$ usually correspond to density above 1.08. We can conclude that the shallow temperature in the ocean is high, the density is small and the data is large. But low temperature, high density and low data volume in deep oceans. Based on the distribution characteristics of density data points, the distribution of density data points in the upper ocean indicates that the density difference is large, the distribution is uneven, and the possibility of existing pycnocline is higher than that of discrete data points. Therefore, we select the data from 0–500 depth range as training set and test set, thus simplifying the experimental data.

Then, we need to preprocess the raw data before making prediction of machine learning. Since the original data only include temperature, salinity and pressure, we need to calculate the required density data according to the density calculation formula described in the previous chapters. The format after data processing is consistent with the right side of Table 1.

In the end, according to the introduction of the second chapter, we list the selection of the density gradient, based on the following Table 2:

### 3.2. The Prediction of Pycnocline was Realized Based on Python

#### 3.2.1. The Ratio of Training Set Was Determined by Polynomial Regression

Before using Kernel-SVM algorithm to fit the data set, we should divide the data set into training set and test set according to a certain proportion. However, how to determine the ratio is related to the accuracy of the final prediction.

**Figure 6:**
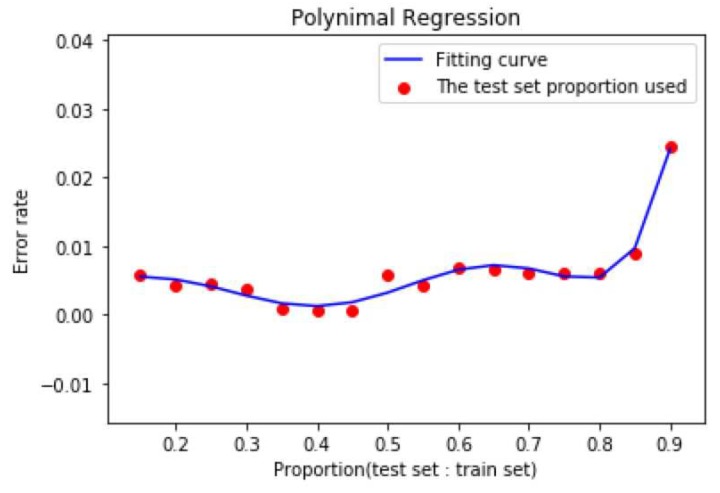
Polynomial regression curve related to the ratio of test set to training set and error rate.

In order to determine the ratio between the training set and the test set, we compare the experimental data with different proportions and obtain the prediction accuracy corresponding to different proportions. Here, we introduce the concept of confusion matrix (error matrix to represent the decision error and accuracy. Confusion matrix expresses the relationship between total data and error data by matrix. Each column of confusion matrix represents the forecast category, and the total number of each column represents the number of data predicted as that category; each row represents the true home category of the data, and the total data of each row represents the number of data instances of the class. In this step, we do not study the influence of super parameters on data in detail. In order to obtain the ratio between training set and test set, we do not set the super parameters of the model in polynomial regression analysis. Then, the obfuscation matrix of the data was inquired in the proportion of different training sets and test sets, and the following Table 3 of prediction accuracy was obtained:

It can be seen that the precision is increasing basically before the ratio is less than 0.45, and the precision decreases after the ratio is greater than 0.45. This shows that the training set is not as simple as the support vector machine algorithm. In order to select a suitable ratio, we select the ratio between test set and training set as independent variable X and the accuracy of polynomial regression for dependent variable y. In order to be more accurate and avoiding overfitting, the polynomial regression curve like the following figure (Figure 6) is obtained by using the power 7 as the best proportion.

It can be clearly seen from the curve that the error rate is between 0.3/0.4/0.45 and 0.45. From the actual measurement, when the ratio is 0.45, the error rate is the smallest and the pycnocline predicted is the most accurate. Considering that the accuracy does not increase monotonously with the scale, this reflects the characteristics of the Kernel-SVM algorithm from the side: we can use less training set data to fit the curve, and we can get very accurate results. If the ratio is too high, the prediction effect is not necessarily better, and the curve will show the phenomenon of fitting. Based on this, we set the ratio of training set to test set to 0.45, that is, 1925 random points out of 3500 points as training set and 1575 points as test set.

#### 3.2.2. Data Smoothing by Spline Interpolation

The basic form of SVM is a hard-spaced classifier, which requires all samples to satisfy the hard-spaced constraint. So when there are noise points in the data set, in order to divide the noise points correctly, the hyperplane will approach the sample of another class, which makes the geometric spacing of dividing the hyperplane smaller and reduces the generalization performance of the model. The data of temperature, salinity used in our experiment are not smooth, which is very likely to increase the error rate of prediction, so in this section we smoothed the data of temperature and salinity.

Firstly, the 0–500 m are divided into 4000 copies, but the 4000 data used in our experiment are not necessarily uniformly distributed. Then we used Python’s spline interpolation function to get a smooth curve. Specifically, we need to find a trinomial polynomial approximating the curve between each pair of data points. The cubic spline function *f*(*x*) is a piecewise cubic polynomial.

(21) $$f\left(x\right)=\left\{\begin{array}{c} f_{1}\left(x\right)\;\;\;\;\;\;\;x_{1}<x<x_{2}\\ f_{2}\left(x\right)\;\;\;\;\;\;\;x_{2}<x<x_{3}\\ ...\\ f_{3999}\left(x\right)\;\;\;\;\;x_{3999}<x<x_{4000} \end{array}\right.$$

**Figure 7:**
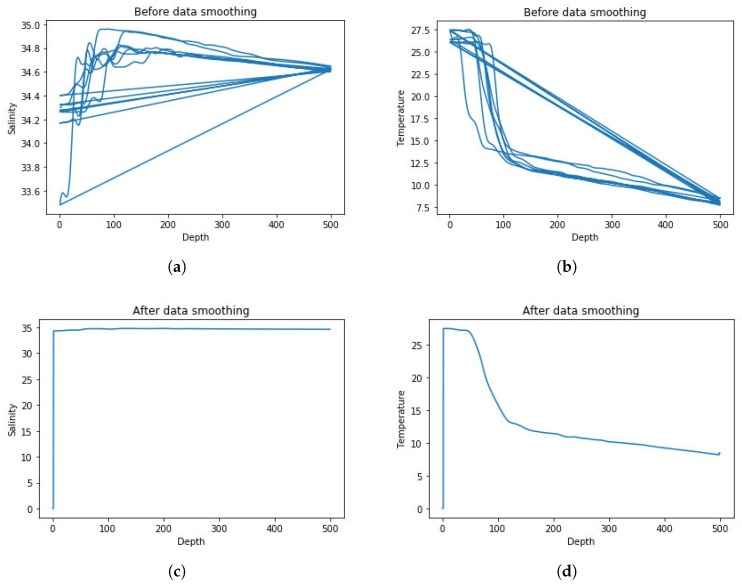
(**a**) salinity data before smoothing. (**b**) temperature data before smoothing. (**c**) salinity data after smoothing. (**d**) temperature data after smoothing.

Figure 7 shows a comparison of temperature and salinity data before and after data smoothing. From the graph before data smoothing, we can conclude that the original data has duplicate values, most likely incorrect values and outliers. We use data smoothing method based on spline interpolation to pre-process the data in the first step, discard these noise points, and improve the accuracy of prediction results.

#### 3.2.3. Use Feature Scaling (FS) to Speed up Gradient Retracting

In the process of training, we find that the depth of the column is of order of 0–500, while the other column data is only 0–50. So in the process of training and fitting, the large eigenvalue will affect the length of the Euclidean distance, which leads to the long running time of the program and the slow convergence process of the gradient. To solve this problem, we preprocess the data set, and use the transform function in the StandardScaler function library of python to scale the features of the data used [33,34]. The following formulas are used to find the center and scale most of the data between −3 and 3, which speeds up the convergence of the gradient.

(22) $$\begin{array}{c} {X_{n}}^{\prime }=\frac{X_{n}-\mu_{n}}{\delta } \end{array}$$

In the above expression (Equation (22)), $X_{n}$ denotes the nth feature, that is, the nth dimension of the characteristic variable X. $\mu_{n}$ denotes the mean value of the feature, that is, the average value of the nth feature of all eigenvector sets, and δ denotes the standard deviation.

We take the depth and density gradient as independent variables and the pycnocline marker as dependent variables. Left side of Table 4 is the test set data that does not use feature scaling. The right side is the test set data that used feature scaling:

#### 3.2.4. Parameter Optimization Process Based on Variable Step Size Grid Search Algorithm

When the SVM classifier is used to establish the prediction model, if the super parameter is not optimized, the default penalty factors are C = 10 and gamma = (1/feature number). However, different penalty factors and kernel function coefficients have great details for the experimental classification results, which even determines whether the classifier is suitable for predicting the data of marine types from the very beginning.Therefore, it is necessary to determine the super parameter C and gamma of the SVM algorithm before establishing the prediction model. At present, the commonly hyperparametric search methods are: grid search, random search and Bayesian optimization. Among all of them, grid search and Bayesian optimization are widely used. Common grid search consumes computing resources, especially when there are many hyperparameters. In 2012, Jack applies Bayesian optimization to machine learning.Bayesian optimization updates the posterior distribution of the objective function by adding sample points to the given objective function. The number of iterations in Bayesian optimization is small, So it saves time. However it is difficult for us to choose Kernel Matrix of Gauss Process. In this paper, we propose an improved grid search to improve the search speed while reducing the computational burden. In the experiment, we used the Bayesian optimization method in [35] to optimize the super-parameters, and compared with the improved grid algorithm. We find that Bayesian optimization is fast,it takes about 71.3315336 seconds. And it iterates 30 times. The improved grid search takes 192.8725636 seconds, and the total number of iterations is 3. But the SVM accuracy rate of Bayesian optimization:98.9% is lower than that of the improved grid search: 99.3%. So we draw a conclusion that the grid search method for complex ocean data variable step size is more suitable for application.

The main idea of the grid search algorithm is to divide the parameters to be optimized (C and gamma) into grids according to equal step size within a fixed range. By traversing the values of all parameters and using K-CV (k-fold Cross validation, we set K as 10 in the experiment, that is, the algorithm of 10 fold). Cross validation is used to obtain the accuracy of the training set under this group of parameters. Finally, the group of parameters with the highest cross-validation accuracy of the training set is selected as the corresponding optimal solution. In order not to fall into the misunderstanding of local optimal solution, we usually increase the optimization range of parameters and reduce the search step infinitely, which will greatly increase the operation and time consumption of optimization. For this reason, a grid search algorithm with variable step size was used to determine the super parameter C and gamma. Based on the traditional grid search algorithm, the optimization process was divided into two steps: coarse search and fine search. First, a preliminary search is carried out by setting a large search step. The optimal solution under the rough search step will obtain the super parameter C and gamma satisfying the local optimal solution. However, if something unexpected happens, such as having different C values and gamma values at the same time, so that the final accuracy rate remains the same, we will choose the group with the larger C value without changing the gamma value too much. Since C (penalty factor) represents the tolerance of the model to the loss caused by outliers, a higher value of C ensures that the accuracy of the prediction model will not be reduced due to the neglect of individual outliers. Starting from the second search, we set the starting point of the search as the optimal solution obtained last time, and the step size as 20 percent of the last step size. The search range changes to the new local optimal solution C and gamma within the range of 5 adjacent steps. Keep going this way until you find the super parameters C and gamma that satisfy the global optimal solution.

In the variable step size grid search, we integrated the standardized operation and classifier SVM in the pipeline. We know that the value range of C and gamma is usually between $10^{-5}$ to $10^{5}$, so at the beginning, we defined the rough search range to be between $10^{-5}$ to $10^{5}$ with a step size of 10. By using the grid search method with variable step size, after the first rough search, the local optimal solution was obtained: C = 1000, gamma = 0.0001. Starting from the second step, the step size changed to 10×0.2. Since the values of C and gamma were different, we extended the range to 5 adjacent steps of C and gamma, that is, from 11 points at the beginning to 16 points. In this way, the global optimal super parameter information can be obtained. The local optimal solution of each step is shown in the following Table 5:

Thus, the optimal solution obtained by the fifth time is the same as that obtained by the fourth time, which means that the local optimal solution is also the global optimal solution (C = 512, gamma = 0.02097152).

#### 3.2.5. Feature Ranking Based on Learning Model

When the density cannot be obtained, it is of great military significance to use other marine data to predict the density thermocline. However, due to the adverse environment on the ocean or the tight time for preparation, we cannot obtain a variety of marine data types in some situations. We should at least first obtain the data that has the largest impact on the density thermocline. For this reason, it is necessary to carry out characteristic weight analysis on characteristic variables [36]. Our specific approach is to build a prediction model based on the super parameters obtained by combining each individual feature and response variable, and then make model analysis for each feature and response variable respectively and obtain the error rate.Finally, the scores of each feature are sorted. The sequence of the obtained characteristic scores is shown in the following Table 6:

Thus, in the case of unknown density, depth (pressure) and temperature are the characteristic variables most closely related to the density thermocline.

#### 3.2.6. The Experimental Results

In this section, we applied the classifier fitted by the training set to the test set and obtained the predicted results of the SVM classifier under the condition that the input was 10 characteristic variables (without density data) and the super parameter was gamma = 2.088, C = 6.833. By comparing the density data obtained by the real calculation with the density skip data obtained by the gradient determination method, we obtain the accuracy rate based on the variable step size GridSearch-SVM model. Finally, we compared this model with the traditional 5VM method (C = 1, gamma = 1/10) and some other related machine learning algorithms. As shown in the following Table 7:

The comparison results of different model in Table 7 shows that the variable step size GridSearch- SVM has the highest accuracy. NuSVC takes the longest computing time and logical regression takes the shortest time. The variable step size GridSearch-SVM ’s calculation time is not particularly short but it guarantees high accuracy. So we think it can be used to predict the location of pycnocline.

**Figure 8:**
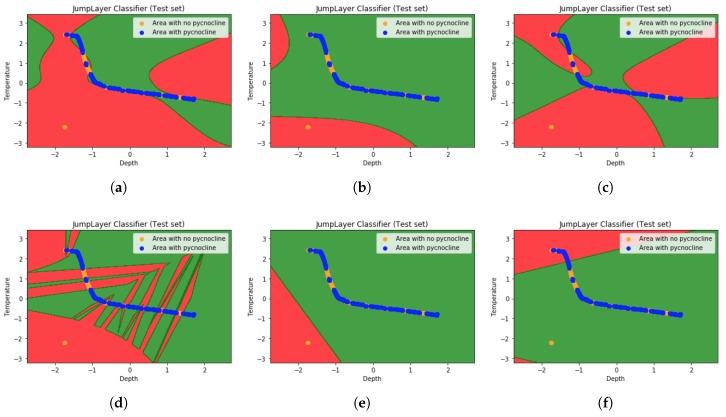
The predicted pycnocline classification image by: (**a**) variable step size GridSearch-SVM method. (**b**) Traditional-SVM method. (**c**) NuSVC method. (**d**) KNN method. (**e**) Logistic regression method. (**f**) Naive bayes (GaussianNB) method.

Since the multidimensional features cannot be shown by the diagram, we selected the maximum depth and temperature of the density shadow as the input features. As shown in the figure below (1, variable step size GridSearch-SVM classification image. 2, Traditional-SVM classification image. 3, NuSVC classification image. 4, KNN classification image. 5, Logistic regression classification image. 6, Naive bayes(GaussianNB) classification image):

We can see from the Figure 8 that the red region is marked as an area where there is no pycnocline. Green region is the region where pycnocline is predicted. The orange and blue dots represent the density data points, where the orange dots indicate that the layer in which the density data is located does not contain pycnocline. And blue indicates that the layer in which the density data is located contains pycnocline. It is not difficult to find that, when some blue data goes to the red area, it means that there is an error between the predicted data and the actual data.

It can be seen from the graph that the accuracy of the variable step size GridSearch-SVM algorithm is higher than other machine learning algorithms when the input is 2 features. We can also draw a conclusion that the location of pycnocline can be predicted in a better way based on the variable step size GridSearch-SVM algorithm when the density data is unknown. In addition to density and depth data, temperature is also an important factor to predict the density thermocline.

## 4. Conclusions and Future Work

In this paper, the characteristics of ocean density varying with longitude and latitude, depth, temperature, salinity, season and so on are summarized, and the distribution characteristics of ocean density are verified from images by matlab. Secondly, combined with the polynomial regression model of machine learning and Kernel-SVM algorithm, the ocean hydrological data is trained and predicted, and good accuracy is obtained. Compared with the traditional choice of pycnocline, using machine learning method can use less known data (such as only known depth and density gradient) to predict pycnocline. We know that in many cases, ocean hydrological data measurements are not easy and some polar regions or regions with bad weather are more difficult to detect oceanic hydrological data. If we can get the classified data with very high prediction rate, we can reduce the dependence on unknown data when we judge the existence of pycnocline. To some extent, it is convenient for marine underwater research and marine military activities.

In the future, with the explosive growth of marine big data, making efficient use of oceanic big data is still an important research direction. We will continue to explore faster, more accurate and more complex prediction models for ocean hydrological data with a combination of in-depth learning and integrated learning. 

## Figures and Tables

**Table 1:** The original Argo data and pretreatment data type.

Original Column	Original Feature	Pretreatment Column	Pretreatment Feature
Column 1	Pressure (dbar)	Column 1:	Depth (m)
Column 2	Corrected Pressure (dbar)	Column 2:	Pressure (dbar)
Column 3	Quality on Pressure	Column 3:	Temperature (degree Celsius)
Column 4	Temperature (degree Celsius)	Column 4:	Salinity (PSU)
Column 5	Corrected Temperature (degree Celsius)	Column 5:	Density (kg/m3)
Column 6	Quality on Temperature	Column 6:	Longitude
Column 7	Salinity (PSU)	Column 7:	Latitude
Column 8	Corrected Salinity (PSU)	Column 8:	Year
Column 9	Quality on Salinity	Column 9:	Month
Column 10	Flag of all	Column 10:	Day
		Column 11:	Gradient
		Column 12:	Flag

**Table 2:** Selection of density gradient.

Depth	Gradient	Pycnocline
0 < d < 200	G ≥ 0.1	1
0 < d < 200	G < 0.1	0
d > 200	G ≥ 0.015	1
d > 200	G < 0.015	0

**Table 3:** Prediction accuracy of test set to training set.

Selection Proportion	Test Set	Training Set	Error Rate
0.10	350	3150	0.00287
0.15	525	2975	0.00575
0.20	700	2800	0.00430
0.25	875	2625	0.00457
0.30	1050	2450	0.00382
0.35	1225	2275	0.00082
0.40	1400	2100	0.00071
0.45	1575	1925	0.00064
0.50	1750	1750	0.00575
0.55	1925	1575	0.00417

**Table 4:** Use feature scaling(FS) to normalized test set.

Depth Unused FS	Density Gradient Unused FS	Depth Used FS	Density Gradient Used FS
470	0.045172	1.50892	−0.292717
291	0.051387	0.265468	−0.0483674
64	0.044187	−0.882707	−0.227177
23	0.048328	−1.31104	−0.331443
322	0.045762	−1.59164	−0.168635
36	0.044061	0.481618	−0.26952
432	0.275660	−1.50439	−0.336397
140	0.044861	1.24806	−0.341036
127	0.046271	−0.77959	−0.304944
284	0.042845	−0.874775	−0.249508

**Table 5:** The process of obtaining hyperparameters.

Iteration Times	Step	Parameter: C	Parameter: gamma	Accuracy Rate	Model Score
1	10	1000	0.0001	99.3%	0.995203741214
2	2	50	0.0002	99.3%	0.9925757837
3	0.4	6.833	2.088	99.3%	0.9925757837

**Table 6:** Feature ranking based on score.

Feature Score	Feature Name
0.943	Depth
0.753	Pressure
0.376	Temperature
−0.417	Salinity
−0.51	Day
−0.519	Year
−0.534	Latitude
−0.535	Month
−0.542	Gradient
−0.547	Longitude

**Table 7:** Accuracy comparison of different algorithms.

Model Type	Parameter Setting	First Kind of	Second Kind of	Accuracy Rate	Computational Time
		Misjudgement	Misjudgement		
variable step size GridSearch-SVM	C = 6.833, gamma = 2.088	15	3	99.3%	0.0149986
Traditional-SVM	C = 1, gamma = 0.1	3	17	98.70%	0.0465037
NuSVC	gamma = 0.1	23	23	97.1%	0.1594989
KNN	auto	9	4	99.1%	0.0270001
Logistic regression	auto	3	21	98.48%	0.011999
Naive bayes(GaussianNB)	auto	416	5	73.3%	0.02050018
